# Pulmonary Vein Isolation for Atrial Fibrillation: Comparison of a Very High-Power Very Short-Duration (vHPvSD) Ablation Protocol versus a Hybrid Ablation Approach—Procedural and Mid-Term Outcome Data

**DOI:** 10.3390/jcm13102879

**Published:** 2024-05-13

**Authors:** Alexander Baumgartner, Martin Martinek, Michael Derndorfer, Georgios Kollias, Peter Ammann, Helmut Pürerfellner, Sebastian Seidl

**Affiliations:** 1 Faculty of Medicine, Johannes Kepler Universität Linz, 4020 Linz, Austria; alexander-baumgartner@gmx.at; 2 Department of Cardiology, Ordensklinikum Linz GmbH, 4020 Linz, Austria; martin.martinek@ordensklinikum.at (M.M.); michael.derndorfer@ordensklinikum.at (M.D.); georgios.kollias@ordensklinikum.at (G.K.); helmut.puererfellner@ordensklinikum.at (H.P.); 3 Department of Cardiology, Kantonsspital St. Gallen, 9007 St. Gallen, Switzerland; peter.ammann@kssg.ch

**Keywords:** atrial fibrillation, pulmonary vein isolation, very high power very short duration, hybrid ablation, procedural data, long-term outcome

## Abstract

**Background:** Pulmonary vein isolation (PVI) using radiofrequency ablation (RFA) is a standard-of-care treatment in the rhythm control strategy of symptomatic atrial fibrillation (AF). Ablation protocols, varying in the power and duration of energy delivery, have changed rapidly in recent years. Very high-power very short-duration ablation (vHPvSD) is expected to shorten procedural times compared to conventional ablation approaches. However, the existing data suggest that this might come at the cost of lower first-pass isolation rates, a predictor of poor ablation long-term outcomes. This study aims to compare a vHPvSD protocol to a hybrid strategy, in which the power and duration of the energy transfer are adapted depending on the anatomical location. **Methods:** We retrospectively analyzed procedural and outcome data from 93 patients (55 vHPvSD vs. 38 hybrid) scheduled for de novo pulmonary vein isolation. A vHPvSD ablation protocol (90 Watt (W), 4 s) was compared to a hybrid protocol using vHPvSD on the posterior wall and 50 W HPSD (high-power short-duration) ablation guided by the Ablation Index along the remaining spots. **Results:** Ablation times were significantly shorter in the vHPvSD cohort (5.4 min. vs. 14.2 min, *p* < 0.001), thus resulting in a significant reduction in the overall procedural duration (91 min vs. 106 min, *p* = 0.003). The non-significant slightly higher first-pass isolation rates in the vHPvSD cohort (85% vs. 76%, *p* = 0.262) did not affect freedom from AF 6 months after the procedure (83% vs. 87%, *p* = 0.622). **Conclusions:** vHPvSD helps in shortening the PVI procedural duration, thus neither affecting first-pass isolation rates nor freedom from atrial tachyarrhythmia recurrence at 6 months after the index procedure.

## 1. Introduction

With a lifetime risk of one in three individuals, AF represents the most common sustained cardiac arrhythmia globally [1]. Being directly linked to heart failure, stroke, cardiovascular mortality, and a decreased quality of life, it is associated with substantial morbidity and mortality, thus portending a major public health concern. The management of AF consists of better symptom control, being either based on rate control or rhythm control strategies. Interventional procedures, typically a catheter-based isolation of the pulmonary veins, are a crucial component of the rhythm control strategy in contemporary patient management. Despite the emergence of several alternative technologies using different energy sources and catheter designs, point-by-point ablation using radiofrequency energy has remained the gold standard for interventional AF treatment to date [2,3,4]. Ablation protocols in radiofrequency ablation, varying in the power and duration of energy delivery, have been adapted many times in the recent past. Duytschaever and his colleagues established a standard workflow for pulmonary vein isolation in 2017, with the aim of enclosing the pulmonary veins (PVs) with contiguous and optimized radiofrequency ablation lesions, using a moderate energy setting between 20 and 40 W and an Ablation Index (AI) of ≥400 at the posterior wall and ≥550 at the anterior wall, with an inter-lesion distance (ILD) of ≤6 millimeters (mm) [5]. Especially in comparison to novel competing energy sources, the hereby resulting relatively long ablation times impose constraints on the quantity of ablation procedures that can be performed per day. This fact, and the considerable number of PV reconnections following conventional PVI, has increased interest in exploring higher power applications over a shorter duration—known as high-power short-duration (HPSD) ablation protocols—with power settings up to 50 W, using the same AI target values. Very high-power very short-duration (vHPvSD) ablation, applying even higher energy over a predefined duration (most studies using 90 W over 4 s, with only a minority of studies using 70 W over 5–7 s, without using an AI target value), has become increasingly influential on clinical practice lately. Due to the biophysical characteristics of vHPvSD ablation, by reducing conductive heating and increasing resistive heating, the lesions created appear to be shallower but wider [6]. This strategy aims to reduce procedural times and additionally may enhance PVI safety by minimizing collateral tissue damage [3,7,8]. However, in comparison to conventional approaches that apply lower power over a longer duration, recent investigations have also shown less favorable outcomes in vHPvSD protocols, such as lower first-pass isolation rates and higher acute reconnection rates [3,7]. This study aimed to compare a novel hybrid ablation approach combining 90 W vHPvSD lesions at the posterior region and 50 W HPSD lesions at the anterior region of the left atrium with an established 90 W vHPvSD protocol.

## 2. Materials and Methods

### 2.1. Study Population

This observational registry-based study investigated procedural and mid-term outcome data taken from two independent centers retrospectively, in a non-randomized manner.

Consecutive patients with drug-refractory symptomatic paroxysmal or persistent atrial fibrillation, who underwent de novo radiofrequency AF ablation at either the Ordensklinikum Linz Elisabethinen or the Kantonsspital St. Gallen between January 2022 and December 2022, were included in the data analysis. Baseline characteristics, including information on demography, AF characteristics, comorbidities, and medication, were assessed before ablation for each patient included in the data analysis. The study was designed and executed in accordance with the ethical standards set out in the Declaration of Helsinki. The study protocol was submitted to the local ethics committee. All patients included were informed by the attending physician prior to receiving treatment and gave their consent for their data to be collected in the registry. Patients who declined to allow the acquisition of their data in the clinic’s internal ablation registry were excluded.

Considering these criteria, a total of 93 patients were incorporated in the data analysis. The assignment of the patients to their respective ablation protocol was non-randomized. The patients were treated by 1 of 7 attending electrophysiologists, each of whom had been performing catheter-based ablation procedures for at least 5 years, with a minimum number of ablations of 150 cases per operator per year. All ablation procedures were evenly distributed between the operators. Two operators from the Kantonsspital St. Gallen and one operator from the Ordensklinikum Linz Elisabethinen performed all their procedures using the hybrid approach, while the remaining operators exclusively used the vHPvSD protocol. The treatment method, either vHPvSD or hybrid ablation, was determined randomly, depending on a predefined allocation to a specific timeslot assigned to one of the operators.

Out of the 93 patients, 55 were treated with the vHPvSD protocol, while 38 underwent ablation using the hybrid approach.

### 2.2. Ablation Protocols

The vHPvSD protocol was defined as creating lesions with constant energy delivery settings of 90 W over 4 s in a temperature-controlled mode (target temperature 60 °C, flow rate 8 mL/min, automatic termination of energy delivery at a cut-off temperature of 65 °C). The irrigation flow rate delays the energy application for a minimum of 2 s before and 4 s after each RF application. The very close protocol was utilized in all vHPvSD cases, aiming for an inter-lesion distance (ILD) of 3–4 mm anteriorly and 5–6 mm posteriorly. In the hybrid protocol, the energy application was adjusted based on the anatomical location. Lesions using 90 W over 4 s were performed on the posterior left atrial wall and Ablation Index (AI)-guided 50 W lesions on the remaining areas (50 W, AI 550 anterior and 50 W, AI 400 superior and inferior). The hybrid protocol used an ILD of ≤6 mm without any adjustments based on the anatomical location. The VisiTag^TM^ module (Biosense Webster, Irvine, CA, USA) was employed in all procedures using 50 W lesions to ensure stable catheter positioning and the automated tagging of ablation lesions. The following VisiTag^TM^ settings for filter thresholds were used: To ensure catheter position stability, the minimum time was set at 3 s and the maximum range between lesions at 3 mm. Considering the force-over-time feature of the VisiTag^TM^ module, the time was set at 25%, with a minimum force of 3 g. Impedance drop and target temperature were not taken into account. vHPvSD procedures were performed without the support of VisiTag^TM^.

### 2.3. Ablation Procedure

Within one day prior to the ablation, all patients underwent either contrast-enhanced computed tomography or transesophageal echocardiography, to screen for an intracardiac thrombus and to obtain a detailed understanding of the left atrial (LA) anatomy. No patients were excluded due to anatomical variants of the left atrium. An uninterrupted anticoagulation scheme with non-vitamin K oral anticoagulants (NOACs) was recommended for all ablation procedures. The international normalized ratio (INR) target in patients that received vitamin K antagonists was between 2.0 and 3.0. For intraprocedural anticoagulation, unfractionated heparin was used to achieve an activated clotting time (ACT) above 300 s. In the absence of contraindications, deep conscious sedation using a combination of midazolam, propofol, and fentanyl was administered, according to a physician-led, nurse-administered protocol. A small subset of patients at a high risk of sedation-related complications underwent general anesthesia. Venous access was achieved by triple puncture of the femoral vein. In order to place the mapping catheter (CARTO 3 and LASSO or PENTARAY, Biosense Webster, Diamond Bar, CA, USA) and the ablation catheter (QDOT MICRO catheter and nGEN generator, Biosense Webster, Irvine, CA, USA) in the left atrium, a single puncture was made through the interatrial septum using a steerable sheath (AGILIS, Abbot, Minneapolis, MN, USA). In patients who exhibited AF at the beginning of the procedure, cardioversion was performed to allow mapping in sinus rhythm once an ACT above 300 s was recorded. Contact force (CF) was measured continuously, aiming for a CF between 10 and 40 g, preferably 10–20 g. After completion of the procedure, its effectiveness was verified by placing the mapping catheter into the pulmonary vein ostia to demonstrate the entry block. Exit block testing was performed by positioning the ablation catheter distal to the circumferential lesion, while applying an adequate CF (a minimum force of 3 g) as displayed by the orientation of the force vector. Pacing for the exit block was performed in a point-by-point fashion (comparable to the lesion creation process) by pacing with 10 milliampere and a pulse width of 2 ms.

First-pass isolation was defined as a composite of both the entry and exit block, which needed to be achieved in each PV and at both carina levels. Whenever first-pass isolation could not be achieved in the vHPvSD protocol, touch-up applications were performed using the settings from the 50 W protocol. Gaps in the posterior wall in the hybrid group were treated identically. Whenever an additional ablation line at the cavotricuspid isthmus needed to be drawn in either one of the groups, 50 W lesions with an AI of 400 were used. At the end of the procedure, a transthoracic echocardiography was performed to rule out pericardial effusion. A figure-of-eight suture and a pressure bandage were used to prevent femoral bleeding. The pressure bandage was removed after 6 h and the figure-of-eight suture on the following day.

### 2.4. Postablation Monitoring and Follow-up

Follow-up visits, including a 12-lead electrocardiogram and 24 h Holter-ECG examinations, occurred at 3 months, 6 months, and 12 months after the index procedure or in case of self-declared arrhythmia-based symptoms. The patients were free to choose whether they preferred to have their follow-up visits at any of our two institutions or at their referring doctor. In accordance with current recommendations, a post-procedural blanking period of 3 months was observed.

### 2.5. Outcomes

#### 2.5.1. Primary Endpoints

The first primary endpoint was the influence of the respective method on the overall procedural duration—the time between the initial puncture of the femoral vein and the removal of the last catheter.

The second primary endpoint was defined as the recurrence of AF during the first 6 months after ablation, excluding the blanking period. Any standard 12-lead ECG recording or a single-lead tracing > 30 s showing a heart rhythm with no discernible repeating P waves and irregular RR intervals was counted as AF recurrence.

#### 2.5.2. Secondary Endpoints

Other procedural parameters (ablation time, fluoroscopy time, number of RF lesions, first-pass isolation rate, and complications) were defined as secondary procedural endpoints. The safety endpoint was defined as the incidence of adverse events occurring within 30 days after the ablation procedure, including death, atrio-esophageal fistula, myocardial infarction, stroke or other systemic thromboembolism, transient ischemic attack, phrenic nerve injury, pericarditis, and major vascular access complication or bleeding with the need for surgical or interventional repair.

### 2.6. Statistical Analysis

Nominal parameters were described using absolute and relative frequencies and compared using either the Chi-squared test or Fisher’s exact test in an inferential analysis. Metric data were presented as the mean and standard deviation (SD), while inferential statistics were performed using Student’s *T*-test or the Mann–Whitney *U* test. A test for normality was performed using the Shapiro–Wilk and the Kolmogorov–Smirnov tests. For AF recurrence rates, a Kaplan–Meier estimator was calculated using log-rank testing for statistical significance.

The global significance level α_g_ was defined as α_g_ = 0.05. As two hypotheses were being tested, the significance level for each individual hypothesis was adjusted using the Bonferroni correction. The calculation of the α-correction resulted in an individual significance level of α_I_ = 0.025. All *p*-values of the individual tests below 0.025 were considered significant. All statistical analyses were performed using the statistics software SPSS (IBM, Armonk, NY, USA).

## 3. Results

### 3.1. Patient Characteristics

Overall, a total of 93 patients were enrolled from January 2022 to December 2022. Baseline characteristics are shown in Table 1. A total of 55 patients (mean age 63.5 ± 8.7 years; 69.1% male; 70.9% paroxysmal AF) were treated with the vHPvSD protocol. A total of 38 patients (mean age 63.7 ± 9.4 years; 76.3% male; 57.9% paroxysmal AF) underwent AF ablation with the hybrid approach. The statistical comparison between both groups unveiled one significant difference within the baseline characteristics. The occurrence of congestive heart failure was significantly higher in the hybrid cohort (5% vs. 26.3%; *p* ≤ 0.004). All the remaining evaluated characteristics showed no significant differences between both groups.

### 3.2. First Primary Endpoint

With a similar percentage of procedures performed under continuous sedation (78% vs. 73%, *p* = 0.476), the primary procedural endpoint—total procedural duration—was significantly shorter in the vHPvSD cohort (91 ± 23 min vs. 106 ± 25 min; *p* = 0.003). Details are shown in Table 2.

### 3.3. Second Primary Endpoint

Outcome data were assessed as described in chapter 2.4. Due to a loss-of-follow-up rate of 14.5% and 18.4%, the study population was reduced to 47 patients in the vHPvSD group and 31 patients in the hybrid cohort. During a mean follow-up duration of 10 months (10.2 ± 4.2 months vs. 10.2 ± 4.1 months; *p* = 0.897), atrial tachyarrhythmia recurrence was documented in 8 of 47 patients (17%) in the vHPvSD cohort and in 4 of 31 patients (13%) in the hybrid cohort. No significant difference in AF recurrences was observed between both groups (*p* = 0.622). At the follow-up date, antiarrhythmics were taken by 4.3% versus 11.4% of the participants (*p* = 0.217). Table 3 presents the outcome data in more detail.

### 3.4. Secondary Endpoints

As expected, through the different ablation protocols in conjunction with the almost identical amount of RF ablation lesions (81 ± 16 vs. 82 ± 18; *p* = 0.718), the ablation time in the vHPvSD cohort was significantly shorter (5.4 (4.5–8.4) minutes vs. 14.2 (10.3–18.2); *p* = < 0.001) compared to the hybrid cohort, as was the fluoroscopy time (5.7 (3.8–8.5) minutes vs. 8.6 (6.0–12.0); *p* = 0.002).

Although statistically non-significant, the first-pass isolation rate was slightly higher in the vHPvSD cohort (85% vs. 76%, *p* = 0.262). Acute PV reconnections were primarily observed at the right carina level, especially in its posterior region. The other main localization with a high occurrence of reconnections was the left atrial ridge, which separates the left atrial appendage from the left superior pulmonary vein. All pulmonary veins were successfully isolated at the end of all index procedures.

The one complication that occurred in a patient treated with the hybrid protocol was a delayed cardiac tamponade, which was not detectable in the routinely performed postinterventional echocardiography and became clinically apparent on the day after the procedure, requiring a percutaneous pericardiocentesis. Due to the delayed onset of the pericardial effusion, the exact pathomechanism and its potential relation to the procedure remain unclear.

**Figure 1:**
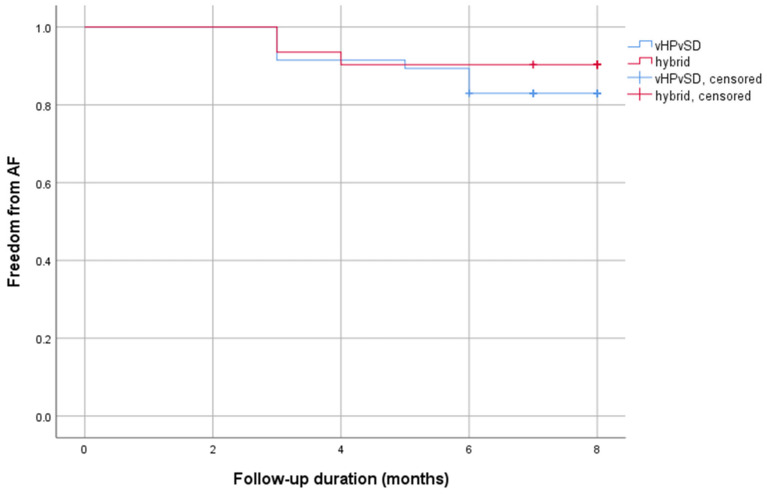
Kaplan–Meier curve AF recurrence. Abbreviations: AF = atrial fibrillation.

Figure 1 displays a Kaplan–Meier estimator illustrating the occurrence of AF relapses in relation to the time since the intervention within the first six months post-ablation.

## 4. Discussion

This study represents one of the initial analyses of procedural and outcome data for a hybrid ablation approach that combines 90 W and 50 W lesions, in comparison to a vHPvSD technique. Our retrospective data analysis revealed the following three major findings:

(1) As expected, the vHPvSD protocol significantly reduced ablation times; this time saving did further result in a statistically significant reduction in overall procedural times.

(2) Contrary to our expectations, the hybrid ablation approach neither led to higher first pass isolation rates nor a reduction in AF recurrences during the follow-up period.

(3) In experienced hands, vHPvSD ablation appears to be as safe as the hybrid ablation approach, as it did not result in an increased incidence of periprocedural complications.

Since the introduction of single-shot technologies, procedural times for PVI have significantly decreased. In these approaches, the median overall procedural times have been reported to be around 40 min [9]. To compete with these technologies in the future, and also from an economical point of view, the shortening of procedural durations will be crucial. The combination of a higher energy delivery over a shorter duration in the vHPvSD protocol, with an almost identical number of lesions in both groups, resulted in statistically significant differences concerning ablation times, which further transferred into shorter overall procedural times and shorter fluoroscopy times. The time saving in the ablation times was expected and is consistent with other recent studies comparing vHPvSD protocols with HPSD approaches [3,7,8].

Although the impact of complete electrical isolation after the first encirclement of the pulmonary veins on clinical outcomes is not entirely clear, it is generally considered a predictive parameter for durable lesion quality and is associated with favorable long-term outcomes [10]. The short application of high energy on cardiac tissue in vHPvSD ablation leads to an increase in resistive heating and a decrease in conductive heating [6]. Based on these biophysical characteristics, the lesion geometry of vHPvSD differs from conventional radiofrequency-based ablation lesions [11]. Results from in vivo animal studies comparing lesion characteristics between a catheter allowing temperature- and flow-controlled ablation (QDOT MICRO^TM^) in the setting of vHPvSD ablation and a standard power-controlled ablation (Thermocool Smarttouch^®^ SF) showed that the former produced larger, shallower, and more homogeneous ablation points [12]. vHPvSD lesions showed a reduction in both macroscopic and microscopic gaps and were less hemorrhagic, leading the authors to conclude that vHPvSD ablation might produce better and more durable lesions in the atria, resulting in a better encirclement and an improved contiguity [12]. Magnetic resonance imaging (MRI) at 3 months after vHPvSD ablation proved durable and transmural ablation lesions, with homogeneous and contiguous scar formation [13].

Lozano-Granero et al. and Bourier et al. demonstrated that lesions created with the vHPvSD (90 W 4 s) protocol using the same catheter (QDOT MICRO^TM^) were smaller, shallower, and thinner than those created with a 50 W AI-based (400 and 550) protocol [14,15]. These findings would support a hybrid ablation approach to increasing the first-pass isolation rate, by placing the potentially more reliable 50 W lesions in areas of increased tissue thickness and catheter instability, while the shallower vHPvSD lesions may be more suitable on the thin posterior wall to avoid esophageal injury [16]. Our hybrid cohort did not show the—based on the previous statements—expected higher first-pass isolation rate compared to the vHPvSD group. The lack of significance in the first-pass isolation rate favoring the hybrid ablation approach might be explained by several reasons. The first-pass isolation rate in our vHPvSD cohort is in the upper range but consistent with other recent trials and a meta-analysis on this topic [17,18,19], indicating the value of highly experienced operators with expertise in the concept of vHPvSD ablation protocols. Both the duration at the ablation point and the stability of the catheter represent crucial components for the effective transmission of radiofrequency energy. The utilization of a vHPvSD ablation protocol decreases the necessary contact time and, consequently, the required duration of a stable catheter position. This may result in a more efficient method of energy transmission.

Kuno et al. proved that general anesthesia (GA) improves catheter stability during pulmonary vein isolation [20]. Performing all PVI procedures under GA may potentially increase first-pass isolation rates even further.

Our own experiences and other recent studies have shown that reducing the inter-lesion distance (ILD) from 5–6 mm to 3–4 mm for anterior areas in vHPvSD ablation improves outcomes, by addressing an important shortcoming of vHPvSD protocols [8,17,21]. The mechanism behind this reduction of the ILD was derived from a pre-clinical study which showed that the application of two vHPvSD lesions at the same spot significantly increased tissue temperature and caused a 40% deeper lesion formation [22]. Considering this, the authors recommended an ILD of 3–4 mm for 90 W vHPvSD ablation [22]. Most of our acute PV reconnections were found in the posterior region of the right carina level, an area with decreased catheter stability, and at the left atrial ridge, an anatomical location where the atrial tissue is known to be thicker compared to other areas of the left atrium. These flaws in our vHPvSD cohort are in line with former studies [7]. Both protocols, the vHPvSD and the hybrid, used identical 90 W 4 s lesions, with an ILD of 5–6 mm at the posterior aspect of the right carina level. Therefore, significant differences between the two cohorts regarding acute reconnections in the posterior area would be unexpected. Based on these considerations and our findings of a high first-pass isolation rate in the vHPvSD cohort, reducing the ILD from 5–6 mm to 3–4 mm for anterior lesions seems to improve the shortcomings of vHPvSD ablation at the level of the left appendage ridge. Apart from the catheter stability and tissue thickness, these two anatomical areas are known to be more likely to have epicardial connections.

Our sample size is too small to conclude any safety efficacy, yet within our vHPvSD cohort, we did not observe any complications. This finding is in line with a systematic review where vHPvSD ablation resulted in a relatively low incidence of adverse events, without any significant differences in terms of cardiac perforation compared to conventional ablation protocols [19].

With more than 80% of patients showing freedom from AF at six months after the ablation procedure, both analyzed ablation protocols showed satisfactory clinical outcomes. Although AF recurrence is the gold standard measure of PVI efficacy, it is still not the most objective endpoint of procedural success and is certainly not the only predictor of a successful long-term outcome, as there is not always a direct relationship between PV reconnection and AF recurrence. Nevertheless, we consider this finding to be relevant, as it demonstrates the non-inferiority of vHPvSD ablation compared to a hybrid ablation approach.

## 5. Limitations

This study displays certain limitations. The retrospective, non-randomized, observational design of the study distinctly reduces its informative value. Due to its design, the study cannot prove causality, is susceptible to confounding and bias, and its results are not generalizable to other centers. Given the relatively small sample size and low incidence of complications, this study was underpowered to evaluate the safety profile of the vHPvSD and hybrid ablation protocols for PVI. With a mean of 10 months, our follow-up duration is shorter than what is usually considered the minimum follow-up time for this type of study. Among the baseline characteristics, the significantly higher occurrence of congestive heart failure in the hybrid group (26.3% vs. 5%; *p* = 0.004) is the only relevant inhomogeneity that should be considered a limitation. We included both patients with paroxysmal and persistent atrial fibrillation. Determining AF recurrence based on a Holter ECG is clearly inferior to an implantable loop recorder-based continuous rhythm-monitoring follow-up.

## 6. Conclusions

vHPvSD ablation for PVI resulted in shorter procedural times, while not adversely affecting first-pass isolation rates or atrial tachyarrhythmia recurrences at 6 months after the index procedure. Taking this into account, the hybrid approach did not show any advantages, considering the increased risk of intraprocedural complications due to longer interventions, as well as higher doses of sedatives and X-ray radiation. However, to draw definitive conclusions on the two ablation methods, conducting prospective, multi-center, randomized, controlled trials with a large sample size will be necessary. Until then, it does not seem essential to adjust the power settings according to the ablation region.

## Figures and Tables

**Table 1:** Baseline characteristics study population.

Characteristics	vHPvSD (N = 55)		Hybrid (N = 38)		
					*p*-Value
**Patient Characteristics**					
Age (years, mean ± SD)	63.5	8.7	63.7	9.4	0.927
Male sex (absolute/relative in %)	38	69.1	29	76.3	0.445
BMI (kg/m^2^, median (IQR))	28.4	26.2–32.1	27.4	25.2–32.1	0.410
**Disease Characteristics**					
Paroxysmal AF (absolute/relative in %)	39	70.9	22	57.9	0.194
LVEF (%, median (IQR))	60	50–60	55	50–60	0.237
LA diameter (mm, mean ± SD)	44.1	6.3	45.3	7.5	0.512
CHA2DS2-VASc-Score (median (IQR)	2	1–3	2	1–3	0.455
Creatinine (mg/dL, mean ± SD)	0.99	0.23	1.04	0.23	0.110
GFR (mL/min, median (IQR))	80	66–89	72	62–84	0.151
	**Absolute**	**Relative**	**Absolute**	**Relative**	***p*-Value**
**Concomitant Diseases**					
Arterial hypertension (absolute/relative in %)	31	56.4	18	47.4	0.393
Diabetes mellitus (absolute/relative in %)	4	7.3	4	10.5	0.582
Coronary artery disease (absolute/relative in %)	6	10.9	6	15.8	0.490
Congestive heart failure (absolute/relative in %)	3	5	10	26.3	0.004
**Medication**					
Oral anticoagulation (absolute/relative in %)	55	100.0	38	100.0	
Beta blocker (absolute/relative in %)	28	50.9	26	68.4	0.920
ACE inhibitors (absolute/relative in %)	24	43.6	16	42.1	0.883
ARNI (absolute/relative in %)	4	7.3	5	13.2	0.345
Mineralcorticoid-receptor antagonists (absolute/relative in %)	8	14.5	9	23.7	0.262
SGLT2 inhibitors (absolute/relative in %)	6	10.9	9	23.7	0.100
Diuretics (absolute/relative in %)	8	14.5	12	31.6	0.058
Statins	21	38.2	11	28.9	0.357

Abbreviations: N = number of sample size; SD = standard deviation; IQR = interquartile range; BMI = body mass index; LVEF = left-ventricular ejection fraction; LA = left atrium; GFR = glomerular filtration rate; ACE = angiotensin converting enzyme; ARNI = angiotensin-receptor neprilysin inhibitor; SGLT2 = sodium glucose transporter 2.

**Table 2:** Procedural data.

Parameter	vHPvSD (N = 55)		Hybrid (N = 38)		
					*p*-Value
Procedural duration (min, mean ± SD)	91.0	22.5	106.2	25.0	0.003
Ablation time (min, median (IQR))	5.4	4.5–8.4	14.2	10.3–18.2	<0.001
Fluoroscopy time (min, median (IQR))	5.7	3.8–8.5	8.6	6.0–12.0	0.002
Average contact force (g, mean ± SD)	19.5	3.5	18.4	3.1	0.124
RF ablations (number, mean ± SD)	81	16.4	82	18.0	0.718
	**Absolute**	**Relative**	**Absolute**	**Relative**	***p*-Value**
First-pass isolation (absolute/relative in %)	47	85	29	76	0.262
Complications (absolute/relative in %)	0	0	1	2.6	0.409

Abbreviations: N = number of sample size; SD = standard deviation; IQR = interquartile range; LA = left atrium; GFR = glomerular filtration rate; ACE = angiotensin-converting enzyme; RF = radiofrequency; g = grams, unit of mass.

**Table 3:** Outcome data.

Parameter	vHPvSD			Hybrid			
	Absolute	Relative	SD	Absolute	Relative	SD	*p*-Value
Follow-up data							
General study population	55			38			
Lost to follow-up	8	14.5%		7	16.7%		0.622
Follow-up population	47			31			
FU duration (months, mean ± SD)	10.2		4.2	10.2		4.1	0.897
AF Recurrence at 6 months							
Patients with recurrence > 90d	8	17%		4	13%		0.622
Medication at follow-up							
Antiarrhythmic drugs	2	4.3%		4	11.4%		0.217

Abbreviations: SD = standard deviation; FU = follow-up.

## Data Availability

The data underlying this article will be shared on reasonable request to the corresponding author.
